# Flavodiiron Protein Activity Outcompetes Cyclic Electron Transport When Expressed in Angiosperm *Nicotiana tabacum*

**DOI:** 10.1111/ppl.70453

**Published:** 2025-08-13

**Authors:** Nicholas Rizzetto, Eleonora Traverso, Andrea Sabia, Filippo Fiorin, Carlotta Francese, Livio Trainotti, Tomas Morosinotto, Alessandro Alboresi

**Affiliations:** ^1^ Department of Biology University of Padova Padova Italy

**Keywords:** flavodiiron proteins, photoprotection, photosynthesis, physcomitrium, tobacco

## Abstract

In conditions of excess illumination, alternative electron transport pathways in the thylakoid membranes protect the photosynthetic apparatus against damage from eventual over‐reduction. Two main pathways downstream of photosystem I (PSI) enable alternative electron flow, mitigating PSI acceptor‐side limitation, while contributing to ATP biosynthesis without reducing NADP^+^ to NADPH: cyclic electron transport (CET) and pseudo‐cyclic electron transport (PCET). Flavodiiron proteins (FLV) are crucial enzymes in PCET, found in all photosynthetic organisms but lost during the evolution of angiosperms. The absence of FLV coding sequences in angiosperm genomes raises intriguing questions about their role and function in photosynthetic organisms. Previous studies utilizing heterologous expression have already demonstrated that FLV can indeed function in angiosperms. In this study, *Physcomitrium patens* FLVA and FLVB coding sequences were stably expressed in wild‐type *Nicotiana tabacum* , a model crop species. Transgenic lines exhibited significantly increased PCET rates, with FLV‐dependent electron transport competing for electrons with CET, particularly under sudden increases in light intensity that limited acceptor‐side limitation. These findings indicate that heterologously expressed FLVs are not only active but can also play a critical role in protecting from over‐reduction the photosynthetic apparatus of *Nicotiana tabacum* under fluctuating light conditions.

## 1 Introduction

Photosynthetic organisms utilize sunlight to drive electron transfer from water to NADP^+^, producing NADPH, through the activity of two photosystems (PSII and PSI). This process also generates a transmembrane electrochemical gradient, leading to ATP synthesis. Together, NADPH and ATP sustain carbon dioxide (CO_2_) fixation and several other metabolic reactions (Stirbet et al. 2020).

In natural environments, plants live in dynamic conditions, including dark‐to‐light transitions, variable light intensities, and heterogeneous water and nutrient supply. These factors can disrupt the balance between excitation energy availability and the utilization of photosynthesis end‐products, potentially leading to over‐reduction of the electron transport chain. In the worst‐case scenario, excess electrons may react with oxygen, generating reactive oxygen species (ROS) that can cause irreversible damage to the photosynthetic apparatus (Joliot and Johnson 2011). Mechanisms regulating photosynthesis are crucial to respond to such imbalances, with a major impact on photosynthetic efficiency. Optimizing these mechanisms can also represent a potential strategy to enhance biomass accumulation under field conditions (Kromdijk et al. 2016; De Souza et al. 2022).

Photosynthetic organisms evolved various regulatory mechanisms, including non‐photochemical quenching (NPQ), cyclic electron flow (CEF), and pseudo‐cyclic electron flow (PCEF). CEF and PCEF operate downstream of PSI, decoupling ATP synthesis from NADPH production and protecting PSI from over‐reduction (Alboresi, Storti, and Morosinotto 2019; Ruban and Wilson 2021; Leister 2023).

The two known CEF mechanisms recycle electrons from ferredoxin (Fd) back to the plastoquinone (PQ) pool. The first is mediated by the chloroplast NADPH dehydrogenase‐like (NDH) complex (Joliot and Johnson 2011) while the second involves the PGR5/PGRL1 system, which facilitates electron transport from PSI back to the PQ pool (Munekage et al. 2002; DalCorso et al. 2008). Although PGR5 and PGRL1 proteins are clearly involved in regulating CEF (Munekage et al. 2002; Johnson et al. 2014), the precise mechanism by which the electrons are transferred from PSI acceptors to the PQ pool remains under debate (Nawrocki et al. 2019).

PCEF keeps PSI in an oxidized state by transferring P700‐derived electrons to molecular oxygen, converting it into H_2_O (Messant et al. 2021). Since the electrons originate from H_2_O by the activity of the PSII oxygen‐evolving complex and ultimately reduce oxygen to water through PCEF, this process is also often termed the “water–water cycle.” PCEF contributes to ΔpH generation but does not support NADPH synthesis and is mediated by two distinct mechanisms: the Mehler reaction and flavodiiron proteins (FLVs).

Mehler reaction detoxifies the superoxide anion produced in PSI when molecular oxygen reacts with electrons from Fd or Fe‐S clusters (Foyer and Hanke 2022). Superoxide is first converted into hydrogen peroxide by superoxide dismutase and subsequently scavenged into water molecules by ascorbate‐peroxidase (Asada 2000).

In the stroma, FLVs accept electrons downstream of PSI to reduce molecular oxygen into H_2_O. These proteins belong to the multigenic flavodiiron protein (FDP) family, found across a diverse range of organisms (Folgosa et al. 2018). In photosynthetic organisms, FLVs consist of three functional domains: an N‐terminal metallo‐β‐lactamase with a diiron‐binding center, followed by a domain able to interact with a flavin mononucleotide (FMN) and a final C‐terminal domain with NADPH:flavin oxidoreductase activity (Folgosa et al. 2018; Alboresi, Storti, Cendron, and Morosinotto 2019; Beraldo et al. 2024).

FLVs function as safety valves for PSI over‐reduction in cyanobacteria (Allahverdiyeva et al. 2013), algae (Chaux et al. 2017; Shimakawa et al. 2022), non‐vascular plants (Gerotto et al. 2016; Shimakawa et al. 2017) and gymnosperms (Ilík et al. 2017; Bag et al. 2023). In these organisms, they play a critical role during dark‐to‐light transitions and under fluctuating light intensities when the Calvin‐Benson cycle is inactive and thus cannot fully utilize the reducing equivalents produced by the linear electron transport chain (Storti, Segalla, et al. 2020). Their activity is not detectable during steady‐state photosynthesis, even though their presence may still generate an impact (Traverso et al. 2025).

Mutant lines deficient in FLV accumulation in different species exhibit pronounced light sensitivity when exposed to light fluctuations, highlighting the critical role of FLVs in photoprotection across various organisms (Jokel et al. 2015; Gerotto et al. 2016; Shimakawa et al. 2017). Despite this general role, FLVs were lost during angiosperm evolution (Hanawa et al. 2017; Ilík et al. 2017) and are also absent in some eukaryotic algae, such as diatoms (Shimakawa et al. 2019).

One possible reason why angiosperms have not conserved FLVs may be attributed to the fact that FLV catalyzes a futile reaction that dissipates electrons, which could be detrimental for efficiency in limiting light conditions. This is consistent with the observation that at least if strongly over‐expressed, FLV activity can become detrimental (Traverso et al. 2025). This will be accompanied by the increased efficiency in angiosperms of other electron sinks such as the cyclic electron pathway (Yamamoto et al. 2016), the Calvin‐Benson cycle, and photorespiration (Hanawa et al. 2017; Shikanai and Yamamoto 2017) that could have made the FLV activity less essential and thus dispensable.

Attempts to express heterologous *FLV* genes from cyanobacteria or the moss *Physcomitrium patens* in angiosperms have demonstrated that these proteins can function as electron acceptors downstream of PSI (Yamamoto et al. 2016; Gómez et al. 2018; Wada et al. 2018). In wild‐type *Arabidopsis thaliana* and *Oryza sativa* , PSI is limited on the acceptor side upon light activation and following sudden increases in light intensity. However, expression of *P. patens* FLVA and FLVB in wild‐type plants reduces this limitation, demonstrating that the heterologously expressed proteins are active and contribute to PCEF rates under fluctuating light conditions (Yamamoto et al. 2016; Wada et al. 2018). Similarly, *Nicotiana tabacum* lines expressing cyanobacterial *Flv1‐Flv3* exhibit faster responses during dark‐to‐light transitions (Gómez et al. 2018). Moreover, expression of *P. patens* FLVs has been shown to partially rescue the phenotypes of CEF knock‐out lines (Yamamoto et al. 2016; Wada et al. 2018). No measurable FLV activity was instead detected during steady‐state photosynthesis, and their expression does not significantly impact carbon fixation under constant illumination, suggesting that the contribution of PCEF under steady‐state conditions is negligible (Yamamoto et al. 2016; Wada et al. 2018). While these studies confirm that FLVs are active when expressed heterologously in angiosperms and can act as alternative electron sinks and contribute to the response to light fluctuations, several important questions remain without an answer. A key unresolved issue is whether FLVs provide a measurable advantage in photosynthetic efficiency or stress tolerance under conditions such as fluctuating light, prolonged high‐light exposure, or rapid dark‐to‐light transitions.

To address this point, we expressed *P. patens* *FLVA‐FLVB* in WT *N. tabacum* . Our results confirm not only that FLVs are active in this species and capable of supporting a high flow of electrons but also that the transport rates are comparable to those observed in the native organism *P. patens* (Gerotto et al. 2016; Traverso et al. 2025). We also quantified the electron flow in WT and FLV‐expressing lines, discovering how FLV actively oxidizes P700 alternatively to CEF, supporting the hypothesis that CEF compensates for the absence of FLVs in angiosperms. Additionally, we report for the first time that, by mitigating PSI acceptor‐side limitation, PCEF significantly enhances photoprotection upon exposure to light fluctuations.

## 2 Materials and Methods

### 2.1 Plant Material and Growth Conditions

*Nicotiana tabacum* cv. Samsung NN, WT, and FLV‐expressing plants were sowed on watered filter paper, and after germination, plantlets were transferred to germinators for 1 week; finally, they were planted into single pots for all the analyses. Plants were grown in a growth chamber at 25°C, 45%–65% air humidity under a photoperiod of 16‐h day (100 μmol m^−2^ s^−1^) and 8‐h night. To test photosynthetic efficiency under fluctuating light conditions, 3‐week‐old plants were positioned under an LED lamp (Photon System Instruments; SL 3500) and subjected to 4.5 min at 50 μmol m^−2^ s,^−1^ followed by 30 s at 1000 μmol m^−2^ s^−1^. The photoperiod was 16‐h of fluctuating light and 8‐h night. To increase the stress related to light fluctuation, this treatment was performed in a growth chamber at 16°C.

*Physcomitrium patens* ssp. Gransden WT and *flv* KO (Gerotto et al. 2016). All plants were routinely propagated as previously described on PpNH_4_ rich medium at 24°C under long‐day conditions (16/8 h day/night). For photosynthetic measurements and for plant growth quantification, the individual lines were cultivated photoautotrophically on PpNO_3_ minimal medium (Ashton et al. 1979; Storti et al. 2019).

### 2.2 Molecular Cloning and Plant Transformation

The introduction of the two *P. patens* *FLVA* and *FLVB* genes in *N. tabacum* was achieved by expressing the *FLVA‐2A‐FLVB* sequence (Yamamoto et al. 2016) under the control of the 35S constitutive promoter.

*FLVA‐2A‐FLVB* gene (4182 bp) was subcloned in pDONR221 and then transferred into the pBinAR expression vector (Hofgen and Willmitzer 1988) using the Gateway cloning system to obtain pBinAr‐35S::FLVA‐2A‐FLVB vector. *Agrobacterium tumefaciens* strain LBA4404 was used for the stable transformation of *N. tabacum* plants (Fisher and Guiltinan 1995). Young, healthy, and undamaged leaves were collected from in vitro grown WT tobacco plants and soaked in a suspension of *A. tumefaciens* cells (OD_600_ ~ 0.8) in MS salts without vitamins (4.3 g L^−1^), sucrose (30 g L^−1^), 6‐BAP (1 mg L^−1^) and Gamborg B5 medium (1 mL L^−1^ of 1000× stock solution) for 10 min to allow the infection cycle. Leaves were then placed on TAB1 co‐cultivation medium (MS salts with vitamins 4.4 g L^−1^, sucrose 30 g L^−1^, 6‐BAP 1 mg L^−1^, IAA 0.2 mg L^−1^, plant agar 5 g L^−1^, pH 5.7) and incubated in the dark at 23°C for 48 h. Leaves were then transferred to plates containing TAB2 medium (TAB1 medium supplemented with kanamycin 200 mg L^−1^ and cefotaxime 500 mg L^−1^) and incubated at 23°C with a 16/8 h light/dark photoperiod. The TAB2 medium was refreshed every 15 days until callus formation occurred. Once shoots of 1–3 cm in length emerged from the callus masses, independent clones were cut off and placed in Magenta boxes containing TAB3 medium (MS salts with vitamins 4.4 g L^−1^, sucrose 30 g L^−1^, plant agar 6 g L^−1^, kanamycin 200 mg L^−1^ and cefotaxime 500 mg L^−1^, pH 5.7) for rooting. After ~20 days, plants were analyzed by PCR on genomic DNA to confirm the presence of the transgene and then transferred to soil. The selected T_0_ plants were self‐crossed to obtain the corresponding T_1_ and T_2_ lines. Segregation analysis of the transgene was conducted by sowing pre‐sterilized T_2_ seeds in MS 1/2 medium supplemented with kanamycin 200 mg L^−1^ (Figure S2). The plates were incubated in a growth chamber at 25°C (16/8 h light/dark photoperiod) for 15 days, after which germinated plants, resistant to kanamycin selection, showed green developed cotyledons.

### 2.3 Gene Expression Analysis

RNA was purified from leaf disks with TRI Reagent (Sigma‐Aldrich) and used as a template for cDNA synthesis to verify FLVA, FLVB, and H2A expression. The primers used for PCR are the following: FLVAf: 5′‐GATGCCCCGGGATGGAGACGATGTTGGTG‐3′, FLVAr: 5′‐CACCCTAACATCTGCCTCCT‐3′, FLVBf: 5′‐CTGTCAAAGCCAAGCAACCC‐3′, FLVBr: 5′‐AGGACTTCCATTTGCCCCAG‐3′, H2Af: 5′‐GAGTTACCATCGCAAGCGGA‐3′, and H2Ar: 5′‐ATGCCTTCTCCTCAGCTGCA‐3′.

### 2.4 Immunoblot Analysis

Total extracts were obtained by grinding leaf disks in 150 μL sample buffer (50 mM TRIS pH 6.8, 100 mM DTT, 2% SDS, and 10% glycerol) and centrifuged at 13,000 *g*. An aliquot of the supernatant was used for chlorophyll extraction in acetone 80%. Chlorophyll content was quantified at the spectrophotometer (Cary Series UV–Vis, Agilent Technologies) applying the formula below (Porra et al. 1989):

$${\text{Chls}}_{a+b}\;\left(\mu g\;{\mathrm{mL}}^{-1}\right)=17.76\times A_{646.6}+7.34\times A_{663.6}$$

The supernatant was incubated at 100°C for 1 min before loading into SDS/PAGE. The quantity of samples to load was estimated in terms of equivalents of chlorophyll amount. For immunoblotting analysis, after SDS/PAGE, proteins were transferred to nitrocellulose membranes (Pall Corporation) and hybridized with specific commercial antibodies (PSAA, Agrisera, catalog numbers AS06 172) and homemade polyclonal antibodies (PsaD, γATPase, Cyt f, PsbS, D2, NDH‐H, RuBisCO, and LHCII) and custom‐made anti‐FLVB. The proteins were detected with alkaline phosphatase‐conjugated secondary antibody and colorimetric assay.

### 2.5 Fluorescence and P700 Measurement With Dual‐PAM

The redox changes of P700 were recorded using a Dual‐PAM100 measuring system (Heinz Walz) during 6 s long illumination (2000 μmol m^−2^ s^−1^). Before the measurement, moss samples and tobacco leaves were dark adapted for 30 min. Samples were measured under standard atmospheric conditions as well as anaerobic conditions. Anaerobiosis was induced by incubation of the samples in a plastic box filled with nitrogen‐enriched atmosphere for 15 to 20 min. The oxidation level of P700 was quantified following a repeated Saturating Pulse protocol (rSP) (Shimakawa et al. 2019). Leaves were dark‐adapted for 30 min and then exposed to a first saturating pulse for the determination of the total oxidizable P700 (Pm) and then to 6 SPs (~2000 μmol m^−2^ s^−1^) lasting 1 s applied every 10 s in the presence of low actinic light (~70 μmol m^−2^ s^−1^). The fraction P700^+^/total oxidizable P700 was calculated by integrating the P700^+^ during the last 1 s‐SP, normalized to the Pm. The effects of fluctuating light on photosynthesis were evaluated on 3‐month‐old dark‐adapted plants. The light protocol consisted of 3 cycles of 5 min low actinic light (~60 μmol m^−2^ s^−1^) followed by saturating actinic light for 1 min (~1600 μmol m^−2^ s^−1^). The following PSI and PSII parameters were calculated: Y (I) as 1‐Y (ND)‐Y (NA), Y (NA) as (P_m_‐P_m′_)/P_m_, Y (ND) as (P700_ox_/P_m_), Y (II) as (F_m′_‐F)/F_m′_, 1‐qL as qL = (F_m′_‐F)/(F_m′_ F_0′_) × F_0′_/F, NPQ as (F_m_‐_Fm′_)/F_m′_, where F_0_ and F_m_ represent the minimum and maximum chlorophyll fluorescence levels (Maxwell and Johnson 2000), whereas P_m_ and P_m′_ represent the P700 signals recorded before and after the onset of a saturating pulse (Klughammer and Schreiber 1994). The electron transport rate around PSI and PSII was calculated as follows: ETRI as PAR × ETR‐Factor × P_PS2_/P_PS1+2_ × Y (I) and ETRII as PAR × ETR‐Factor × P_PS2_/P_PS1+2_ × Y (II), where ETR‐Factor represents the sample absorptance and P_PS2_/P_PS1+2_ the distribution of absorbed PAR to PSII.

### 2.6 Spectroscopic Analyses With a Joliot‐Type Spectrometer

Spectroscopic analyses were performed using a Joliot‐Type spectrometer (JTS)‐10 (Biologic). The change in absorption spectra of pigments due to electric potential gradient, known as electrochromic shift (ECS), was used to calculate the relative amount of PSI and PSII and the electron transport rates (ETR). For each sample, ECS was measured after 30 min of dark adaptation at 520 nm and consecutively at 546 nm to subtract the field‐independent contribution. The total number of functional PSI and PSII was determined by a xenon‐induced single flash turnover and was then used to normalize ETR values. Plants were then exposed to 5 min of continuous light (940 μmol m^−2^ s^−1^) and during this time a dark‐interval relaxation kinetic was applied: light was repeatedly switched off to calculate the non‐photosynthetic contribution to ECS. ETR was calculated as the difference between the ECS slope in the light (SL) and the slope calculated in the dark (SD). The same measurements were carried out also in the presence of 3‐(3,4‐dichlorophenyl)‐1,1‐dimethylurea (DCMU). DCMU was used to inhibit PSII and isolate the contribution of ETR around PSI to ECS. Before detection, two 1 cm^2^ leaf samples were detached from each dark‐adapted plant. One of them was incubated for 1 h in paper soaked with DCMU 80 μM and the other one in deionized water as control.

## 3 Results

### 3.1 Isolation of *Nicotiana tabacum* Lines Expressing Physcomitrium Patens FLVA and FLVB Genes

The coding sequences of FLVA and FLVB from *Physcomitrium patens* were introduced into *Nicotiana tabacum* to investigate the role and functionality of these proteins in an angiosperm system. The open reading frame consisted of *FLVA* and *FLVB*, separated by the self‐cleaving 2A‐peptide derived from foot‐and‐mouth disease virus. This construct was designed to guarantee a stoichiometric production of FLVA and FLVB that form an active heterocomplex, a strategy previously validated in *A. thaliana* and *O. sativa* (Yamamoto et al. 2016; Wada et al. 2018). The final FLVA‐2A‐FLVB sequence was cloned under the control of the constitutive 35S promoter and transformed into *N. tabacum* leaf disks, enabling the generation of transgenic tobacco lines for further characterization.

The functionality of FLVs can be assessed by measuring the redox kinetics of P700 in PSI under short‐pulse saturating illumination (2000 μmol m^−2^ s^−1^ for 6 s), as FLVs have been shown to maintain PSI in a safely oxidized state even upon exposition to such high light (Ilík et al. 2017; Shimakawa and Miyake 2018). The reliability of this measurement to evidence FLVs activity is shown by the comparison between WT *P. patens* , natively expressing them, and *flva/b* KO line. The initial P700 oxidation, detected in both WT and *flva/b* KO mosses, resulted from the PSI charge separation event followed by P700 reduction attributed to electrons extracted from water by PSII and reaching PSI (Ilík et al. 2017). During the light pulse, while the WT P700 pool was oxidized again through the FLV‐mediated electron transport downstream of PSI (Figure S1), P700 in *flva/b* KO mosses remained reduced throughout the light exposure (Figure S1). Given the reliability and efficiency of this measurement, monitoring P700 oxidation kinetics was selected as a rapid screening method to identify transgenic *Nicotiana tabacum* clones expressing functional FLVs.

In *N. tabacum* , WT plants exhibited a P700 oxidation profile comparable to that observed in *P. patens* *flva/b* KO lines, with P700 that remained reduced after the initial illumination (Figure S1). On the contrary, some transformed *N. tabacum* lines exhibited a distinct P700 oxidation pattern resembling that of WT mosses, thus suggesting they expressed functional FLVs (Figure S1). Since FLV activity is oxygen‐dependent, an additional control experiment was performed using the same saturating light pulse protocol under anoxic conditions. In WT *N. tabacum* plants, the P700 oxidation profile remained unaltered regardless of oxygen availability (Figure S1). In contrast, the putative FLV‐expressing lines lost their ability to keep P700 in an oxidized state under anoxia, exhibiting WT‐like behavior (Figure S1). This oxygen‐dependent response confirmed the presence and activity of functional FLV proteins in these lines.

Further validation of FLV expression in *N. tabacum* transgenic clones was performed through immunoblot analysis, which detected the accumulation of *P. patens* FLVB in multiple lines. Among the transgenic *N. tabacum* lines showing positive results in both P700 oxidation kinetics and FLV protein accumulation, clones #1, #7, and #8 were selected for propagation to F2 generation and further investigations (Table S1).

**FIGURE 1:**
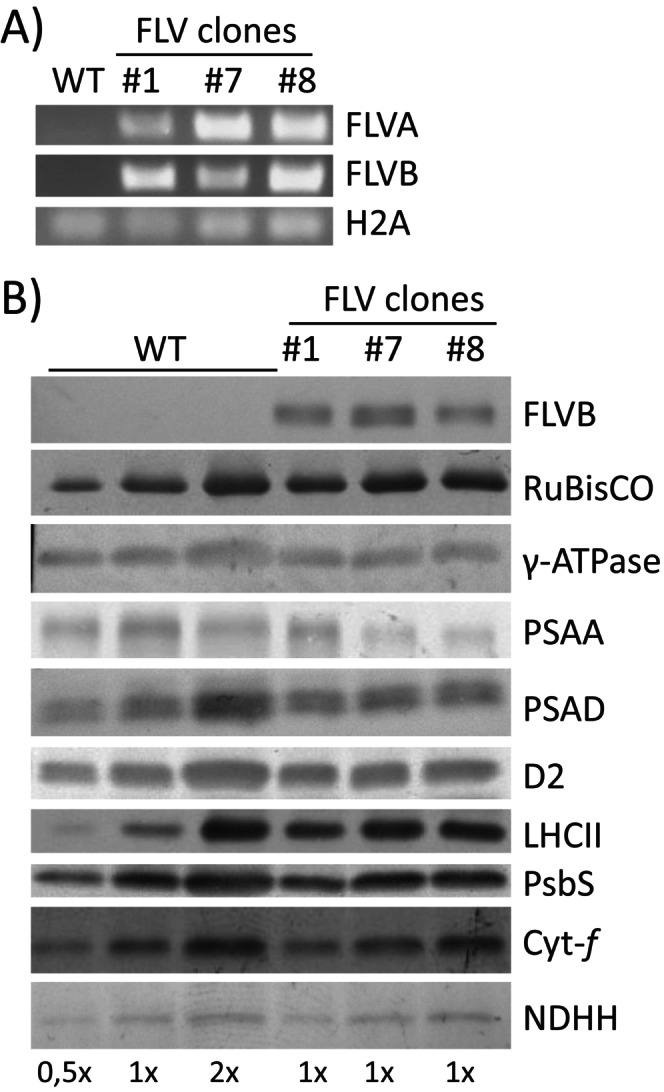
FLV expression and Immunoblot analysis of photosynthesis‐related proteins in *N. tabacum* lines. (A) The expression of FLVA and FLVB was verified via RT‐PCR, H2A histone served as the endogenous control. (B) Immunoblot analysis was performed to detect photosynthesis related proteins in both wild‐type (WT) and transgenic lines of *N. tabacum* expressing FLVA‐2A‐FLVB. The proteins analyzed included FLVB, RuBisCO, γ‐ATPase, PSAA, PSAD, D2, LHCII, PsbS, Cytf, and NDH‐H. The detection was carried out using different chlorophyll equivalents as follows: 1× is equivalent to 2 μg of chlorophylls for the detection of FLVB, γ‐ATPase, PSAA, PSAD, D2, PsbS, Cyt‐f, and NDH‐H. 1× is equivalent to 0.2 μg of chlorophylls for the detection of RuBisCO and LHCII. The amounts 0.5× and 2× represent half and twice the amount of 1×, respectively.

RT‐PCR showed that both FLVA and FLVB genes were expressed in these lines (Figure 1A). Immunoblot analysis confirmed that the accumulation level of FLVB protein was comparable among the selected lines (Figure 1B). To assess whether the expression of FLV proteins impacted the composition of photosynthetic machinery in transgenic *N. tabacum* lines, we analyzed the content of key proteins associated with photosystems and electron transport. The levels of proteins constituting PSII (D2) and PSI (PSAA and PSAD) as well as major antenna proteins (LHCII) showed no evident alterations in FLV transgenic lines as compared to WT. The abundance of Cyt *f* and γ‐ATPase also did not significantly change in FLV lines in respect to WT (Figure 1B). The accumulation of PsbS, the key activator of non‐photochemical quenching (NPQ) of chlorophyll fluorescence at the level of PSII, also remained unchanged between WT and FLV lines (Figure 1B). No significant changes in RuBisCO enzyme or the NDH‐H subunit were detected between WT and FLV lines as well (Figure 1B). Overall, FLVs expression, thus, had no major alterations on the composition of the *N. tabacum* photosynthetic apparatus.

As the three independent lines displayed consistent behavior across all analyses, from here on the overall average data are presented for clarity.

**FIGURE 2:**
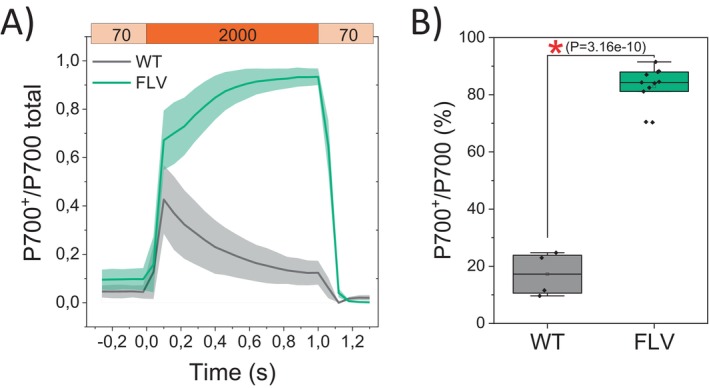
Oxidized P700 fraction in *Nicotiana tabacum* plants. (A) Comparison between wild‐type (WT) and the FLV‐expressing lines, the kinetics of oxidized P700 (P700^+^) during illumination with a short‐pulse light (SP: ~2000 μmol m^−2^ s^−1^, 1 s). WT plants (black) and FLV‐expressing lines (green) were subjected to SP in the presence of a background light of ~70 μmol m^−2^ s^−1^. The relative P700^+^ amount is normalized to Pm, which represents the maximum oxidation level of P700. Standard deviation is indicated as a shadow. (B) P700 oxidation index shown as percentage of P700^+^ over the total P700. The integration of the P700^+^ was calculated as the sum of relative P700^+^ values every 0.3 ms during SP illumination, which was then divided by the maximum integrated area. WT, *n* = 4 ± SD. FLV, *n* = 11 ± SD. A red asterisk indicates statistical significance (one‐way ANOVA).

To validate FLV activity and confirm the initial screening results (Figure S1), we employed a quantitative protocol to determine the fraction of oxidized P700 (P700^+^) relative to the total amount of photo‐oxidizable P700. To this aim, plants were exposed to repetitive 1‐s saturating pulses (rSPs) over a background of moderate light intensity between (Shimakawa and Miyake 2018; Furutani et al. 2022) (Figures 2A and S2A). In WT plants, PSI was oxidized at only 20% of its maximum capacity, while in FLV lines, 80% of PSI reaction centers were kept in an oxidized state during the last 1‐s treatment (Figures 2B and S2B). This result confirmed that *P. patens* FLVs expressed in *N. tabacum* were actively oxidizing P700.

### 3.2 FLV Expression Enhances the Response of *N. tabacum* to Fluctuating Light Conditions

**FIGURE 3:**
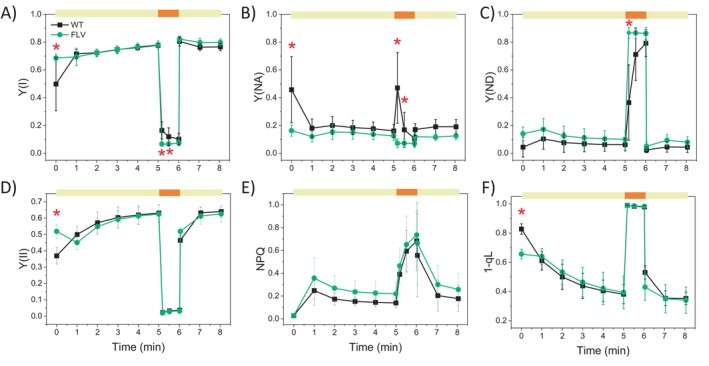
Effect of *P. patens* FLVs on photosynthetic parameters of *N. tabacum* . Photosynthetic efficiency was monitored during fluctuating light cycles for WT plants (black squares) and FLV‐expressing lines (green circles): Y(I) (A), Y(NA) (B), Y(ND) (C), Y(II) (D), Y(NPQ) (E), and 1–qL (F). At time 0, after 40 min of dark adaptation, plants were treated with low actinic light (~60 μmol m^−2^ s^−1^; yellow bars) for 5 min followed by saturating actinic light (~1600 μmol m^−2^ s^−1^; orange bars) for 1 min. This cycle was repeated two more times as shown in Figure S3. Data are averages ±SD, with statistical significance indicated by red asterisks (*p* < 0.01, one‐way ANOVA). WT plants: *n* = 7; FLV lines: *n* = 17.

In *P. patens* plants, FLV activity was indispensable in dark‐to‐light transitions and after a sudden increase in incident illumination (Gerotto et al. 2016; Storti, Segalla, et al. 2020). Consistent results were also found in all FLV‐expressing organisms (Allahverdiyeva et al. 2013; Chaux et al. 2017; Shimakawa et al. 2017). Therefore, we challenged *N. tabacum* plants with three cycles of limiting (low) and saturating (high) light intensities, monitoring different photosynthetic parameters (Figures 3 and S3).

All genotypes showed the same initial F_v_/F_m_ values (WT = 0.78 ± 0.01; FLV lines = 0.77 ± 0.01). During the initial transition from darkness to low light, WT plants had low PSI and PSII yields (respectively Y(I) and Y(II)) that recovered after a few minutes of illumination (Figure 3A,D). While this drop was visible in dark‐adapted plants, it was absent in the following low‐to‐high light cycles (Figure S3), when samples were already light‐adapted. Interestingly, FLV‐expressing lines showed higher Y(I) and Y(II) with respect to WT in this dark‐to‐low light transition (Figure 3A,D). Upon exposure to a stronger light, after 5 min in low light, both Y(I) and Y(II) strongly decreased to ~0.1, as a result of the saturation of photosystem photochemical capacity (Figure 3A,D). In FLV‐expressing lines, there were significant differences in the dark‐to‐light and low‐to‐high light transitions (Figure 3). The cycle was applied consecutively three times (Figure S3). During the third cycle, Y(I) values during the low light period were significantly higher in FLV lines (~0.8) than in WT plants (~0.73) (Figure S3).

PSI activity and efficiency depends on acceptor or donor side limitation, estimated respectively from Y(NA) and Y(ND) parameters. WT plants displayed a steep and transitory Y(NA) increase at each transition from low‐to‐high light intensity (Figures 3B and S3). By the end of the high light treatment, this limitation is recovered, and PSI is only limited from the donor side (Figures 3C and S3). In FLV‐expressing plants, Y(NA) was instead constantly low (Figures 3B and S3), suggesting that FLV was ready to receive electrons from PSI, working as an additional acceptor. Because of the sustained flow of electrons toward FLVs during high light, PSI of transgenic lines was always limited at the donor side (Figures 3C and S3).

Analyzing PSII specific parameters, no significant differences in NPQ (Figures 3E and S3) and in the redox state of plastoquinone pool (1‐qL; Figures 3F and S3) were observed between WT and FLV‐expressing lines for the overall light treatment, but for the 1‐qL parameter at the very beginning of the low light period.

**FIGURE 4:**
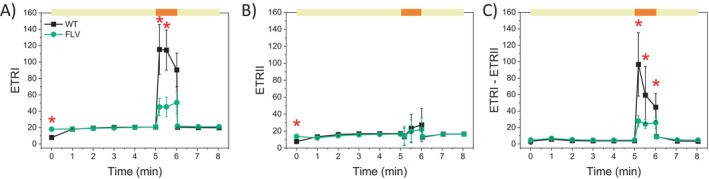
WT *N. tabacum* exhibited higher cyclic electron transport activity compared to FLV‐expressing plants. Photosynthetic electron transport rates in WT (black squares) and FLV‐expressing lines (green circles) were measured during fluctuating light cycles. ETRI (A), ETRII (B), and ETRI‐ETRII (C) as a proxy for cyclic electron transport. At time 0, after 40 min of dark adaptation, plants were treated with low actinic light (60 μmol m^−2^ s^−1^; yellow bars) for 5 min followed by saturating actinic light (1600 μmol m^−2^ s^−1^; orange bars) for 1 min. This cycle was repeated 2 more times as shown in Figure S4. Data are averages ±SD, with statistical significance indicated by red asterisks (*p* < 0.01, one‐way ANOVA). WT plants: *N* = 7; FLV lines: *N* = 17.

Estimation of photosynthetic electron transport rates (ETR) provided further insights into the functional differences between WT and FLV‐transgenic lines, particularly during the transition from low‐to‐high light (Figures 4 and S4). At the PSI level, the estimated electron transport rate (ETRI) in WT plants was significantly higher than in FLV‐transgenic lines within the first few seconds of the low‐to‐high light transition (Figure 4A). At the PSII level, the estimated electron transport rate (ETRII) was instead comparable between WT and FLV‐expressing plants across all light treatments (Figure 4B). This different behavior of the two photosystems suggests that cyclic electron flow (CEF) was also altered. Indeed, its estimation, as the difference between ETRI and ETRII (Huang et al. 2015), suggests that WT plants have transiently higher CEF activity upon high light exposure compared to FLV‐expressing lines (Figures 4C and S4); less evident in FLV‐expressing lines (Figures 4C and S4). It is important to note that these estimations of ETR are based on several assumptions and may be affected by overlapping contributions from other electron pathways influencing P700 redox dynamics (Furutani et al. 2022). Nevertheless, the comparative analysis of genotypes under identical conditions pointed to the presence of differences in photosystems activity between WT and FLV‐expressing lines.

### 3.3 *P. patens* FLVs Increase Photosynthetic Electron Transport at Light Onset in *N. tabacum*

**FIGURE 5:**
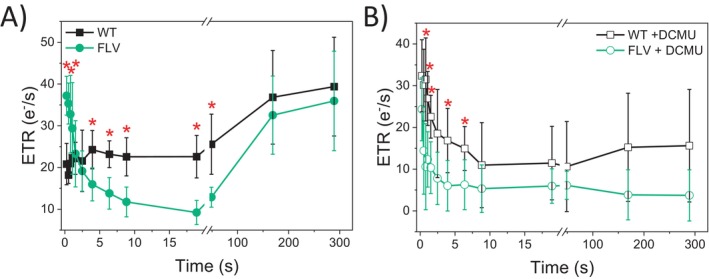
Photosynthetic electron transport in *N. tabacum* WT and FLV‐expressing lines. (A) Total photosynthetic ETR measured in WT (black squares) and FLV‐expressing lines (green circles) at 940 μmol m^−2^ s^−1^ actinic light, calculated from electrochromic shift signal. (B) Cyclic electron transport rate measured in the same samples treated with the PSII inhibitor 3‐(3,4‐dichlorophenyl)‐1,1‐dimethyl urea (DCMU). Data represent average values ±SD, *n* = 6 for the WT, *n* = 17 for FLV lines. Differences between WT and mutant plants were examined by one‐way ANOVA; a red asterisk indicates statistical significance (*p* < 0.01).

Considering the potential limitations of approaches above, photosynthetic electron transport in WT and FLV‐expressing lines was orthogonally evaluated by measuring the electrochromic shift (ECS) of carotenoid absorption. Because ECS reflects changes in the thylakoid membrane potential, it provides a sensitive and non‐invasive indicator of photosynthetic activity. ETR in particular was assessed using dark‐interval relaxation kinetics (DIRK) analysis under actinic light conditions (Sacksteder and Kramer 2000). In dark‐acclimated WT plants, ETR started at ~20 electrons per second and remained stable during the first 20 s of illumination. Successively, ETR steadily increased, reaching a steady state after 4–5 min of illumination when carbon fixation was fully activated (Figures 5A and S5). In *N. tabacum* lines expressing FLV, the ETR was twice that of WT at the onset of actinic light (~40 electrons per second; Figures 5A and S5). This burst of activity was transient, persisting for only a few seconds. Subsequently, the FLV *N. tabacum* ETR decreased to levels even lower than those of the WT. After approximately 5 min of light initiation, when steady‐state photosynthesis was achieved, no statistically significant differences were detected among the genotypes (Figures 5A and S5).

ETR was also estimated in the presence of a PSII inhibitor to assess the CET contribution to the overall transport. WT plants showed high CET activity at the initiation of illumination followed by a gradual decline to a steady level (Figure 5B). Conversely, FLV‐expressing lines consistently exhibited lower CET activity throughout the first seconds of light treatment, reaching a steady state value indistinguishable from WT (Figures 5B and S5).

**FIGURE 6:**
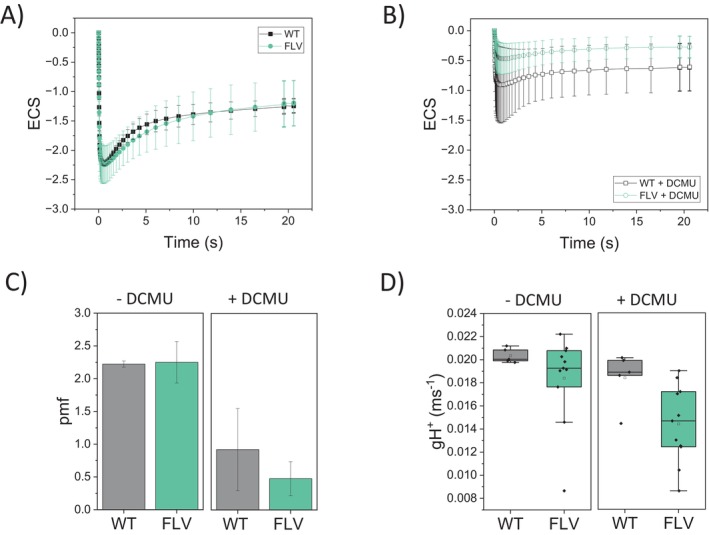
Impact of DCMU treatment on WT and FLV‐expressing lines. (A, B) Electrochromic shift (ECS) signal recorded immediately after light was switched off following 5 min of illumination, in the absence (A) and presence (B) of DCMU. WT is shown in black, and FLV‐expressing *N. tabacum* lines in green. (C) Proton motive force (pmf) calculated from ECS data shown in (A) and (B). (D) Proton conductance (gH^+^) estimated as the inverse of the time constant (1/*τ*) from the initial 300 ms ECS decay after light‐off, based on data in (A) and (B). Data represent mean ± SD; *n* = 5 for WT, *n* = 3 for FLV clone 1, *n* = 4 for FLV clone 7, and *n* = 4 for FLV clone 8. Statistical differences between WT and transgenic lines were assessed using one‐way ANOVA.

During dark recovery after exposition to saturating illumination, both WT and FLV‐expressing lines displayed comparable ECS signals (Figures S6 and 6A) and similar levels of *pmf* (Figures S6 and 6C). Likewise, no significant differences were observed in proton conductance (gH^+^), used as an estimate of ATP synthase activity and proton dissipation efficiency (Figure 6D). This suggests that at steady state, photosynthesis activity is highly similar in WT and FLV‐expressing lines (Figure 6). Interestingly, a similar picture emerges in measurements after a few seconds of illumination (Figure S6) showing that FLV does not impact overall *pmf* generation nor ATP synthase activity.

To further dissect the impact of FLVs in ETR, we treated leaf disks with the PSII inhibitor, 3‐(3,4‐dichlorophenyl)‐1,1‐dimethylurea (DCMU), to suppress linear electron flow and thus specifically quantify CET around PSI (Figures 5B and S5). The inhibitor induced a major reduction in ECS signals (Figure 6B) and *pmf* (Figure 6C) in both WT and FLV‐expressing lines, but no significant difference was detected between the two genotypes. Also, no significant difference in gH^+^ between treated and untreated *N. tabacum* plants and between genotypes was observed (Figure 6D).

### 3.4 FLV Expression Increases Tolerance to Fluctuating Light During Growth

**FIGURE 7:**
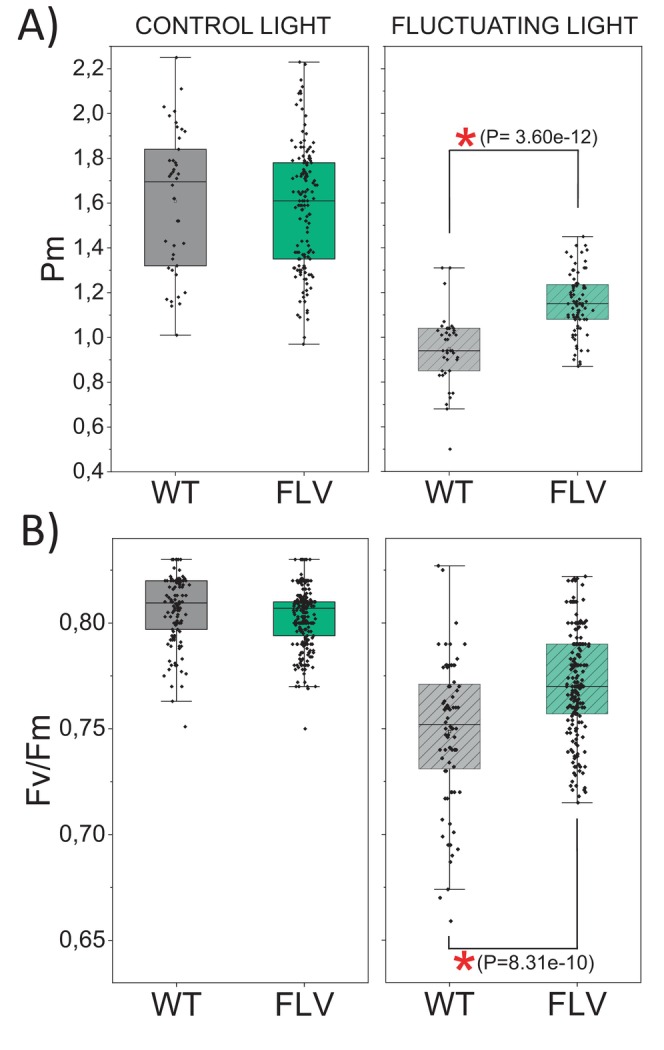
Effect of light regime on PSI and PSII efficiency. Pm (A) and Fv/Fm (B) were measured in dark adapted WT (black) and FLV‐expressing lines (green) grown at different light regimes for 14 days. The left panels show data obtained under control light conditions (100 μmol m^−2^ s^−1^; photoperiod 16/8 h light/dark; 25°C). The panels on the right show data for plants grown under fluctuating light conditions (4.5 min at 50 μmol m^−2^ s^−1^ followed by 30 s at 1000 μmol m^−2^ s^−1^; photoperiod 16/8 h of fluctuating light/dark; 16°C). Data were recorded for each plant once a day. (A) WT, *n* = 3 ± SD. FLV, *n* = 10 ± SD (control light); WT, *n* = 3 ± SD. FLV, *n* = 6 ± SD (fluctuating light). (B) WT, *n* = 11 ± SD. FLV, *n* = 25 ± SD (control light); WT, *n* = 7 ± SD. FLV, *n* = 18 ± SD (fluctuating light). Differences between WT and mutant plants were examined by one‐way ANOVA; a red asterisk indicates statistical significance.

To elucidate the impact of FLVs on photosynthetic activity during plant growth, WT and FLV‐expressing lines of *N. tabacum* were grown under constant control light intensity (CL) and the maximum efficiency of PSII and PSI was monitored as Fv/Fm and Pm (the maximal change in the P700 signal), respectively (Figures 7 and S7). WT and FLV‐expressing lines showed similar values both at the level of Pm (Figure 7A) and Fv/Fm (Figure 7B).

When plants were exposed to fluctuating light conditions (FL; 4.5 min at 50 μmol m^−2^ s^−1^ followed by 30 s at 1000 μmol m^−2^ s^−1^), all genotypes showed reduced Pm and Fv/Fm values compared to their CL‐grown counterparts (Figure 7). However, FLV expressing lines retained significantly higher Pm and Fv/Fm values as compared to WT plants (Figure 7), suggesting a photoprotective effect of FLV during plant growth, making plants more tolerant to light fluctuations.

## 4 Discussion

### 4.1 FLV Has an Intrinsic Ability to Provide Fast Activation of Electron Transport

Upon a sudden increase in light intensity, the photosynthetic electron transport chain reacts rapidly, quickly producing NADPH and ATP. However, the metabolic pathways that consume these energy carriers, such as the Calvin‐Benson cycle, require more time to adjust. This mismatch creates an electron accumulation in the transport chain, particularly downstream of PSI and PSII. This over‐reduction increases the risk of reactive oxygen species (ROS) formation, which can cause oxidative stress and photodamage to key components like PSI (Allahverdiyeva et al. 2013; Gerotto et al. 2016; Chaux et al. 2017; Shimakawa et al. 2017; Bag et al. 2023). The photoprotective effect of FLVs derives from their ability to mitigate imbalances caused by the fast increase in electron transport and the slow regulation of energy‐consuming metabolic processes. Data from different species support the idea that FLVs are essential during the dark‐to‐light transition and during a sudden increase in light intensity to avoid PSI over‐reduction (Allahverdiyeva et al. 2011; Gerotto et al. 2016; Traverso et al. 2025). Due to its capacity of rapidly accepting electrons from PSI, FLVs effectively prevent acceptor side limitation and consequent damage with a protective role. This function has been conserved during the evolution of photosynthetic organisms since it was consistently observed across diverse species, including cyanobacteria (Allahverdiyeva et al. 2013), eukaryotic algae (Jokel et al. 2015; Chaux et al. 2017; Burlacot et al. 2018) and non‐vascular plants (Gerotto et al. 2016; Shimakawa et al. 2017; Storti et al. 2019; Storti, Puggioni, et al. 2020; Storti, Segalla, et al. 2020).

In this study, we introduced FLVs from *P. patens* into the model crop *N. tabacum* plants, a species widely used for engineering various aspects of photosynthesis. These efforts have included modifications to non‐photochemical quenching (Kromdijk et al. 2016), the Rubisco enzyme (Chen, Riaz, et al. 2023), CO_2_‐concentrating mechanisms in chloroplasts (Chen, Hojka, et al. 2023) and the regulation of photorespiration (South et al. 2019).

*P. patens* FLVA/B are successfully expressed and accumulated in *N. tabacum* plants (Figure 1). Their full functionality is confirmed by FLV activity measured at the onset of light activation, as they effectively maintain P700 in a safely oxidized state (Figures 2 and S1) and alleviate PSI acceptor side limitation compared to WT *N. tabacum* (Figure 3B). These effects can be attributed to a significant electron flow toward FLVs, detectable as early as 1 s after illumination begins. A comparable transient increase in ETR is observed in WT *P. patens* but is absent in FLV‐depleted moss lines (Figure 5A; Gerotto et al. 2016). In angiosperms, this role of contributing to ETR upon light fluctuations is rather played by CET (Yamori and Shikanai 2016), whose activity is decreased in FLV‐expressing lines. This may indicate a stronger CEF activation in the WT compared to FLV‐expressing plants.

Interestingly, after 4 s of illumination, an inversion is observed whereby WT plants exhibit higher electron transport rates than FLV‐expressing lines (Figure 5A). Since ETR is derived from ECS signals reflecting membrane potential, this suggests that the contribution of CEF to proton gradient formation exceeds that of PCEF. These results further underscore the complex interplay and competition between CEF and FLV activities (Hubáček et al. 2024).

WT and FLV‐depleted *P. patens* showed comparable electron transport capacity at steady state (Gerotto et al. 2016) where the impact of FLV presence was not detectable. Interestingly, also WT and FLV‐expressing *Nicotiana tabacum* steady state ETR are indistinguishable, thus confirming how FLV contribution decreases when carbon fixation is fully activated (Figure 5A).

These results confirm that FLV is fully active in angiosperms and that no moss‐specific factors are required for its assembly and activity. In particular, the FLV putative electron donors (NADPH and ferredoxin) that have been identified (Peden et al. 2013; Sétif et al. 2020; Beraldo et al. 2024) are clearly present in *N. tabacum* plants. The similar results obtained in *P. patens* and *N. tabacum* also suggested that FLV regulation is intrinsic to the protein or simply dependent on some conserved environmental features, such as light intensity or stroma redox potential and does not require any external specific factor.

### 4.2 FLV Competes With CET for Electrons

Despite their essential role in responding to light fluctuations, FLVs were lost in angiosperms during evolution. Thus, it is hypothesized that FLV activity has been compensated during evolution by an increased CEF and photorespiration activity that ensured enough electron acceptors for PSI to keep in a safe oxidized state (Yamamoto et al. 2016; Hanawa et al. 2017; Fu and Walker 2023). Accordingly, *flv* KO removal from *P. patens* is compensated by a sustained CEF activity compared to WT plants (Gerotto et al. 2016).

However, no negative effects have been identified once reintroduced back into angiosperms in terms of carbon fixation and growth; on the contrary, they can rescue the decreased photosynthetic efficiency of CEF‐depleted *A. thaliana* and rice plants and can improve PSI efficiency under fluctuating light regime (Yamamoto et al. 2016; Wada et al. 2018; Tula et al. 2020).

For the first time, we directly measured the contribution of both alternative electron mechanisms, CEF and PCEF, in WT angiosperm. Our ETRI‐ETRII data as well as ETR measured in the presence of DCMU (Figures 5C and 6B) showed that CEF in FLV‐expressing lines was significantly lower compared to WT, thus implying that when FLVs were present and active, they compete for electrons with CEF. Even though earlier experiments highlighted that FLVs were able to compensate for CEF removal and that FLV expression diminished electron flow toward CEF, their role could not be considered fully overlapping, as WT *N. tabacum* plants exposed to FL revealed significantly lower Pm and Fv/fm in respect to FLV‐carrying lines (Figure 7). PCET competes with CET for electrons and, more broadly, FLVs compete with alternative metabolic pathways such as hydrogen production in (Burlacot et al. 2018; Jokel et al. 2019) and also with the Mehler reaction in *Chlamydomonas reinhardtii* (Pfleger et al. 2024) as well as nitrogen reduction in the heterocysts of diazotrophic bacteria (Ermakova et al. 2014). This competition is consistent with the complementary role and evolutionary development of increased CET, which compensates for the absence of FLV‐mediated photoprotection. While playing a partially overlapping role, these pathways have different kinetic properties, with FLVs providing a rapid sink for excess electrons during transient changes in light conditions while having an undetectable steady‐state activity. On the contrary, CET contribution is slower but provides a steady contribution to ETR (Figure 5) (Tan et al. 2025). The impact of these different kinetics of activation is shown when plants were exposed to fast fluctuations. In these conditions, plants expressing FLVs were more effective in protecting PSI and PSII with respect to WT, relying on CET.

## Author Contributions

**Nicholas Rizzetto:** investigation. **Eleonora Traverso:** investigation, writing – original draft. **Andrea Sabia:** investigation. **Filippo Fiorin:** investigation. **Carlotta Francese:** investigation, supervision. **Livio Trainotti:** conceptualization, supervision. **Tomas Morosinotto:** conceptualization, writing – review and editing. **Alessandro Alboresi:** conceptualization, supervision, writing – original draft, writing review and editing.

## Conflicts of Interest

The authors declare no conflicts of interest.

## Supporting information


**Figure S1:** Redox kinetics of P700 upon dark‐to‐light transition in *Physcomitrium patens* protonemata cells and Nicotiana tabacum leaves. P700 redox kinetics were monitored in vivo during dark‐to‐light transitions in wild‐type (WT) (black line) and flva/b KO lines (red line) of P. patens (upper panel), as well as in WT (black line) and representative transgenic N. tabacum leaves expressing moss FLVs (green line) (middle and lower panels). The kinetics of redox changes were measured in vivo upon exposure of dark‐adapted sample (black bar) to actinic light (2000 μmol photons m^−2^ s^−1^; yellow bar). Light was switched on at time 0 and illumination lasted for 6 s. Samples were either measured under standard atmospheric conditions (top and middle panel) or anaerobic conditions (lower panel). Each curve is the mean of three independent biological replicates, with standard deviation shown as shaded areas.
**Figure S2:** Kinetics of oxidized P700 (P700+) in *Nicotiana tabacum p*lants. (A) Comparison between wild‐type and FLV‐expressing lines, the kinetics of oxidized P700 (P700+) during illumination with a short‐pulse light (SP: 2,000 μmol photons m^−2^ s^−1^, 1 s). Wild‐type plants (black) and three FLV‐expressing lines (FLV clone 1 in green, FLV clone 7 in blue and FLV clone 8 in magenta) were subjected to SP in the presence of a background light of 70 μmol photons m^−2^ s^−1^. The relative P700+ amount is normalized to Pm, which represents the maximum oxidation level of P700. WT, *n* = 4 ± SD. FLV cl. 1 *n* = 4. FLV cl. 7 *n* = 3. FLV cl. 8 *n* = 4. A red asterisk indicates statistical significance between WT and all the three FLV‐expressing lines, analyzed with one‐way ANOVA (*p* < 0.01).
**Figure S3:** *P. patens* FLVs shape electron transport under fluctuating light in *N. tabacum* plants. Effect of fluctuating light on PSI and PSII: Y(I) (A), Y(II) (B), Y(ND) (C), NPQ (D), Y(NA) (E), and 1–qL (F) in the WT (black squares) and three independent lines expressing FLV proteins (green circles for clone 1, blue circles for clone 7 and magenta circles for line 8). At time 0, after 40 min of dark adaptation, plants were treated with low actinic light (60 μmol photons m^−2^ s^−1^; yellow bars) for 5 min followed by saturating actinic light (1,600 μmol photons m^−2^ s^−1^; orange bars) for 1 min. This cycle was repeated 2 more times. Data represent average values ± SD, *n* = 7 for the WT, *n* = 6 for FLV lines clone 1, *n* = 5 for FLV lines clone 7 and *n* = 6 for FLV lines clone 8. Differences between WT and mutant plants in the saturating/limiting light cycles were examined by one‐way ANOVA; a red asterisk indicates statistical significance between WT and all the three FLV‐expressing lines (*p* < 0.01).
**Figure S4:** *P. patens* FLV repress cyclic electron transport in *N. tabacum* plants. Effect of fluctuating light on ETRI (A), ETRII (B) and ETRI‐ETRII (C) as a proxy for cyclic electron transport of WT plants (black squares) and two independent lines expressing FLV proteins (green circles for clone 1, blue circles for clone 7 and magenta circles for line 8). At time 0, after 40 min of dark adaptation, plants were treated with low actinic light (60 μmol photons m^−2^ s^−1^; yellow bars) for 5 min followed by saturating actinic light (1600 μmol photons m^−2^ s^−1^; orange bars) for 1 min. This cycle was repeated 2 more times. Data represent average values ±SD, *n* = 7 for the WT, *n* = 6 for FLV lines clone 1, *n* = 5 for FLV lines clone 7 and *n* = 6 for FLV lines clone 8. Differences between WT and mutant plants in the saturating/limiting light cycles were examined by one‐way ANOVA; a red asterisk indicates statistical significance between WT and all the three FLV‐expressing lines (*p* < 0.01).
**Figure S5:** Photosynthetic electron transport in *N. tabacum* WT and FLV‐expressing lines. (A) Total photosynthetic ETR measured in WT (black squares) and FLV‐expressing lines (green circles for clone 1, blue circles for clone 7 and magenta circles for line 8) at 940 μmol photons m^−2^ s^−1^ actinic light, calculated from electrochromic shift signal. (B) Cyclic electron transport rate measured in the same samples treated with the PSII inhibitor 3‐(3,4‐dichlorophenyl)‐1,1‐dimethyl urea (DCMU). Data represent average values ±SD, *n* = 6 for the WT, *n* = 5 for FLV lines clone 1, *n* = 6 for FLV lines clone 7 and *n* = 6 for FLV lines clone 8. Differences between WT and mutant plants were examined by one‐way ANOVA; a red asterisk indicates statistical significance (*p* < 0.01).
**Figure S6:** Impact of FLV expression in *N. tabacum* pmf generation. (A) ECS (Electro‐Chromic Shift) signal after the light is switch off after 5 s of illumination (WT in black and FLV‐*N.tabacum* in green). (B) Total pmf calculated from ECS data presented in (A). Data represent average values ±SD, *n* = 2 for the WT, *n* = 2 for FLV lines clone 1, *n* = 2 for FLV lines clone 7 and *n* = 2 for FLV lines clone 8. Differences between WT and mutant plants were examined by one‐way ANOVA; a red asterisk indicates statistical significance (*p* < 0.01).
**Figure S7:** Effect of light regime on PSI and PSII efficiency. Pm (A) and Fv/Fm (B) were measured in dark adapted WT (black) and FLV‐expressing lines (FLV clone 1 in green, FLV clone 7 in blue and FLV clone 8 in magenta) grown at different light regimes for 14 days. The left panels show data for plants grown under standard light conditions (100 μmol photons m^−2^ s^−1^; photoperiod 16 h of light and 8 h of dark; 25°C). The panels on the right show data for plants grown under fluctuating light conditions (4.5 min at 50 μmol photons m^−2^ s^−1^ followed by 30 s at 1000 μmol photons m^−2^ s^−1^; photoperiod 16 h of fluctuating light and 8 h of dark; 16°C). Data were recorded for each plant once a day. (A) WT, *n* = 3. FLV, *n* = 3 for clone 1, *n* = 3 for clone 7, *n* = 4 for clone 8 (standard light); WT, *n* = 3. FLV, *n* = 2 for clone 1, *n* = 2 for clone 7, *n* = 2 for clone 8 (fluctuating light). (B) WT, *n* = 11. FLV, *n* = 8 for clone 1, *n* = 6 for clone 7, *n* = 11 for clone 8 (standard light); WT, *n* = 7. FLV, *n* = 6 for clone 1, *n* = 6 for clone 7, *n* = 6 for clone 8 (fluctuating light). Differences between WT and mutant plants were examined by one‐way ANOVA; a red asterisk indicates statistical significance (*p* < 0.01).
**Table S1:** Analysis of T2 generation of FLV transgenic lines. FLV transgenic lines #1, #7, and #8 were tested for resistance to kanamycin. Resistant lines were then tested for the presence of FLVA gene in the genome and for FLV activity by measurement of redox kinetics of P700 upon dark‐to‐light transition.

## Data Availability

The data that support the findings of this study are available from the corresponding author upon reasonable request.
